# Lymphocytic interstitial non-HIV-related pneumonia in pediatrics: a case report

**DOI:** 10.3389/fped.2023.1307607

**Published:** 2024-01-16

**Authors:** Andrea Dionelly Murillo Casas, Diana María Duarte Dorado, Manuela Olaya Hernández

**Affiliations:** ^1^ Facultad de Ciencias de la Salud, Servicio de Alergología Pediátrica, Universidad ICESI, Cali, Colombia; ^2^ Departamento de Pediatría, Servicio de Alergología e Inmunología Pediátrica, Fundación Valle de Lili, Cali, Colombia; ^3^ Departamento de Pediatría, Servicio de Neumología Pediátrica, Fundación Valle del Lili, Cali, Colombia

**Keywords:** lymphocytic interstitial pneumonia, autoimmunity, immune dysregulation, inborn errors of immunity, case report

## Abstract

Lymphocytic interstitial pneumonia (LIP) in pediatric patients without human immunodeficiency virus (HIV) infection remains a poorly characterized and enigmatic disease. Immunological dysregulation, mutations in the COPA gene, and increased morbidity and mortality have been reported in these patients. We present a case of LIP in a pediatric patient without HIV infection. This patient was infected with human T-lymphotropic virus type 1 (HTLV-1) and required right lower lobectomy with pathological findings compatible with lymphocytic interstitial pneumonia. In addition, bronchiectasis, dermatological involvement, and malnutrition were documented. However, no autoimmune disease, polymyositis, myelopathy, or opportunistic infections were found. There were no abnormalities in cellular and humoral immunity. A genetic study identified heterozygous mutations in the SCNN1B, FCHO1, and IL7R genes using single exome sequencing of coding and splicing regions. Although these heterozygous variants are not reported to be aetiological for LIP or diagnostic for the patient's congenital immunodeficiency, we believe they are associated with the severe lung damage seen in the patient's case.

## Introduction

Lymphocytic interstitial pneumonia (LIP) is an uncommon pulmonary lymphoproliferative disorder with an unknown prevalence (1, 2). It is often associated with autoimmune diseases (such as Sjögren's syndrome, rheumatoid arthritis, systemic lupus erythematosus), inborn errors of immunity (common variable immunodeficiency, dysgammaglobulinemia), and viral infections (especially HIV, Epstein–Barr virus). It can also be part of other pulmonary disorders like follicular bronchiolitis, nodular lymphoid hyperplasia, and granulomatous and lymphocytic interstitial lung disease (GLILD) (1, 3, 4).

The clinical presentation of non-HIV-associated LIP is characterized by chronic respiratory symptoms, including persistent cough, dyspnea, fever, and recurrent episodes of pneumonia. These symptoms may manifest early, persist throughout childhood, or worsen as lung function declines. Growth retardation, severe malnutrition, cyanosis, and hypoxemia requiring supplemental oxygen are often associated (1, 2, 5). The findings of the pathology study from histochemical and immunohistochemical studies reveal infiltrates primarily composed of mature and immature polyclonal CD4+ and CD8+ T lymphocytes (1, 2, 6). There is a notable predominance of CD8+ T lymphocytes within the interstice, extending to interlobular and alveolar septa. Additionally, plasma cells and histiocytes may also be detected. Septal thickening represents a prevalent characteristic, typically unrelated to fibrosis and sparing blood vessels (7–9). However, in the later disease stages, architectural loss, fibrosis areas, and bronchiectasis may become evident in nodular formations (6, 7).

In the case of bronchiectasis, it is important to rule out cystic fibrosis (CF) as well as primary ciliary dyskinesia as a cause of bronchiectasis that is not related to CF (10, 11). Evaluating concomitant conditions, such as primary immunodeficiency disorders and autoimmune disease, and performing genetic studies are critical aspects of the assessment (1, 10, 11).

The optimal management of LIP requires an integral evaluation and a multidisciplinary approach (1, 10–13). Currently, randomized studies evaluating treatment options are lacking. Among the pharmacological approaches, systemic corticosteroids have been the most used. Additionally, the utilization of inhaled corticosteroids and bronchodilators alone or in combination with hydroxychloroquine and azithromycin has been described (1, 11, 12). Additionally assessing pulmonary function is crucial for determining prognosis and the extent of pulmonary involvement (1, 14–16).

General management entails respiratory support measures through pulmonary rehabilitation, addressing secondary infections, and in severe cases of pulmonary involvement, lung transplantation may be warranted (1, 11, 12, 16). The prognosis varies, mortality rates tend to be high when complications arise from the management, including pulmonary fibrosis, bronchiectasis, or lymphoma development (1, 3, 17).

In this report, we present a case of a patient from southwestern Colombia with a clinical diagnosis of non-HIV-related LIP. The patient was presented with bronchiectasis and severe chronic pulmonary disease.

## Case report

A patient from southwestern Colombia cared for by the State Children's Service, was first seen by our service at the age of 9 years. The exact details of his medical history were not known, but there were reports of a history of cough, fever, and recurrent pneumonia since the age of infancy. At the age of 4 years, she underwent a right lower lobectomy due to complicated pneumonia and bronchiectasis.

**Figure 1:**
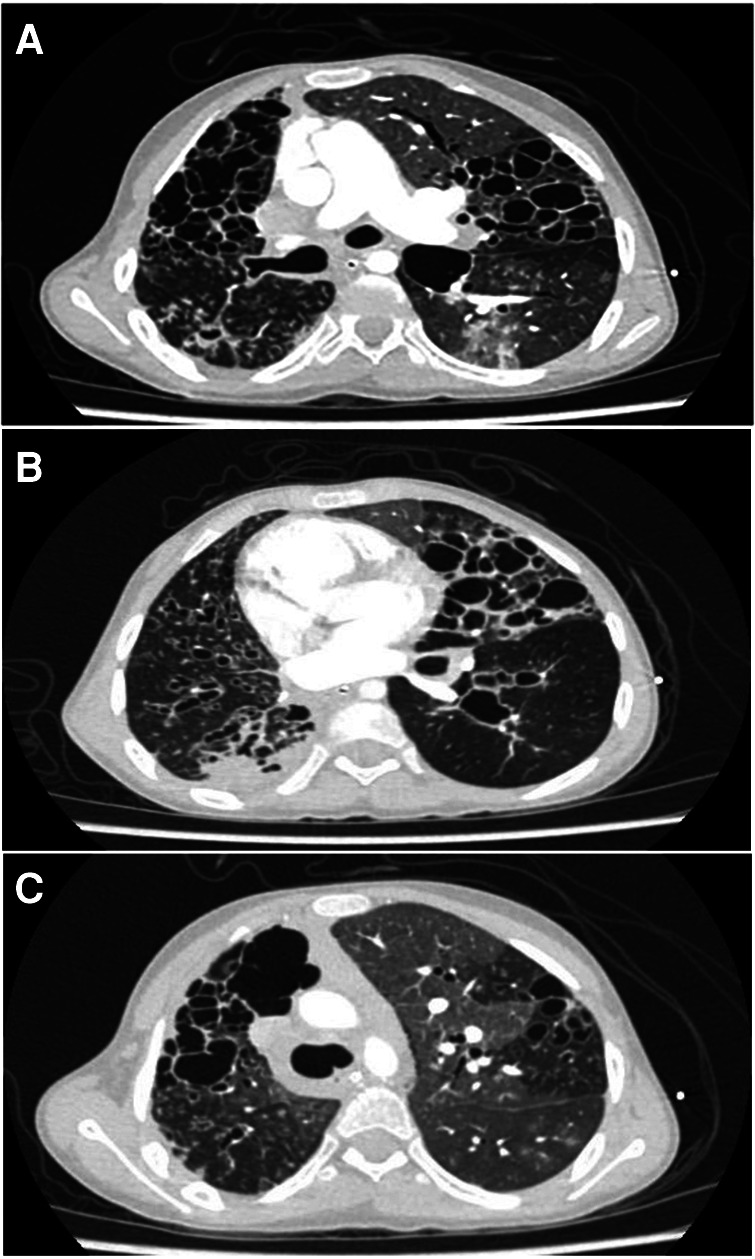
(**A–C**) Correspond to fragments of the high-resolution thoracic computed tomography. Multiple bronchiectasis, varicose and cylindrical, with bronchial thickening, some nodular opacities with twinning pattern and others with ground-glass opacities, mosaic pattern and septal thickening.

**Figure 2:**
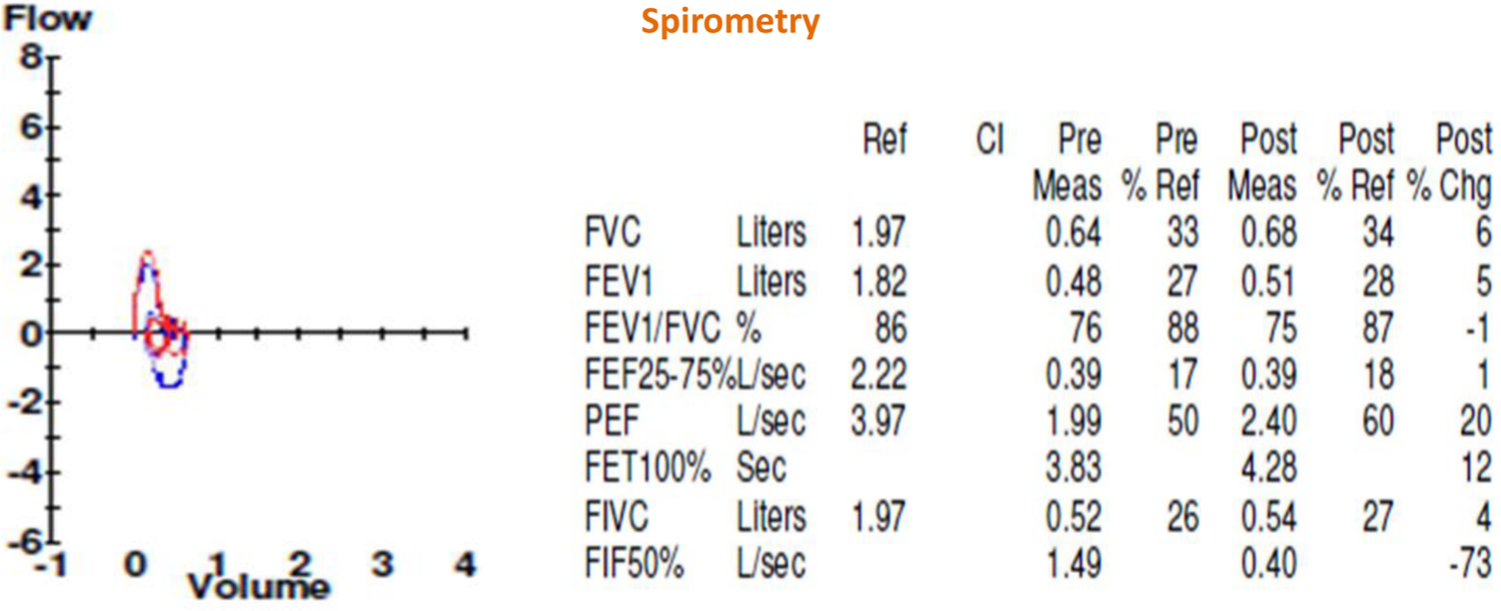
Shows pre- and post-bronchodilator spirometry.

**Figure 3:**
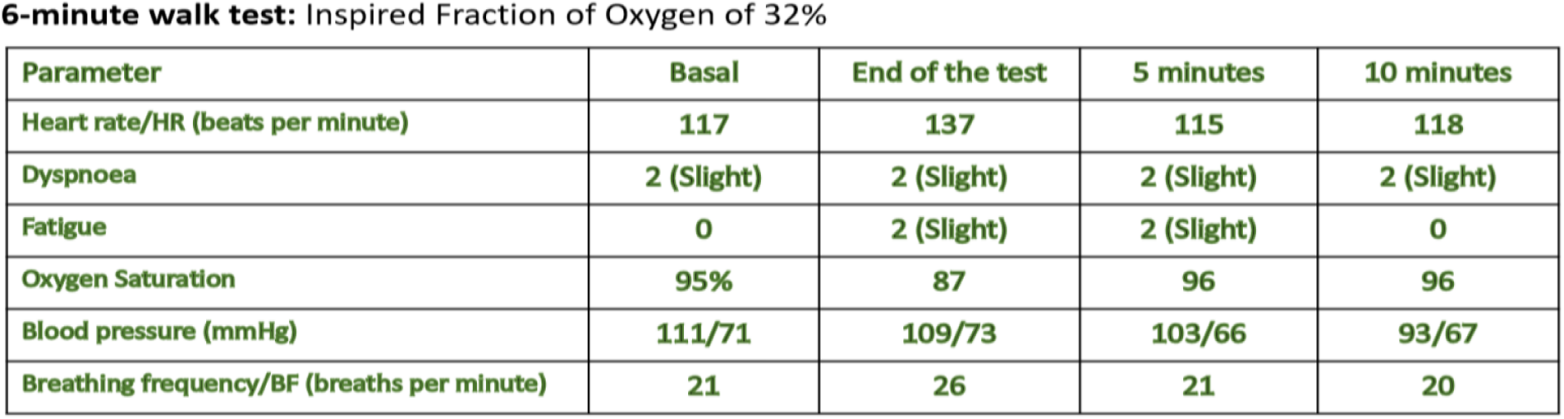
Corresponds to the six-minute walk test. Once the patient was stabilized, lung function was assessed using these tests. The spirometric study showed a restrictive compromise with a very severe reduction of FEV1, normal FVC/EFV1 ratio. In the 6-min walk test, the patient was only able to walk at 7% of the predicted value.

A multidisciplinary approach was essential to establish the diagnosis. In our institution, she required management at the intensive care unit (ICU) due to multilobar pneumonia and respiratory failure. A bronchoalveolar lavage revealed the presence of H. influenzae and S. aureus. While there was no support for fungal or tuberculous infection. The patient exhibited severe bronchiectasis with loss of lung parenchyma (Figures 1A–C). Furthermore, severe chronic malnutrition, muscle atrophy, dermatological involvement, and significant damage to pulmonary function (Figures 2, 3).

Tests for HIV, pulmonary tuberculosis, and *α*1-antitrypsin deficiency returned negative results. Genetic analysis ruled out cystic fibrosis. The patient, however, tested positive for Human T lymphotropic Virus type 1 (HTLV-1). No autoimmune disease, lymphoma, polymyositis, myelopathy, or other opportunistic infection was documented. There were no abnormalities in cellular and humoral immunity. Bronchoalveolar lavage (BAL) flow cytometry showed NK lymphopenia (1.5%) without CD4+ T-lymphocyte involvement with increased CD8+ T-lymphocytes (inversion of CD4+/CD8+ T-lymphocyte formula with a value of 0.8). Histological and immunohistochemical examination of the pathology of the excised lobes revealed findings compatible with lymphocytic interstitial pneumonia and did not show changes consistent with primary ciliary dyskinesia.

Massive parallel single exome sequencing with coding and splicing region analysis revealed a first nucleotide variant c.2005C>T, heterozygous in exon 14/14 of the ENST00000307331 SCNN1B gene transcript. The variant was found in a conserved region at the protein level, p.Arg669Cys. Another nucleotide variant c.2401G>A was found in heterozygosity in exon 24/27 of ENST00000252771 FCHO1 transcript. In a non-conserved region, the change at the protein level p.Val801Ile was found. Lastly, nucleotide variant c.1022G>A, heterozygous in exon 8/8 of IL7R gene transcript NM_002185.5. The protein change p.Gly341Glu was found in a non-conserved region.

Following the initial assessment, the patient underwent comprehensive supportive management, encompassing targeted bronchiectasis therapy, high-dose pulse with methylprednisolone, and a structured pulmonary rehabilitation program. Despite these interventions, the patient's pulmonary function continued to demonstrate severe impairment with an improvement of FVC and FEV 1 of only 9% over the year. Consequently, the medical team initiated discussions regarding the potential suitability and advisability of a lung transplantation procedure, considering the patient's clinical condition and prognosis. This critical juncture necessitated a thorough evaluation of the risks and benefits associated with transplantation, involving a multidisciplinary approach for a well-informed decision regarding the optimal course of action for the patient.

## Discussion

LIP not associated with HIV infection in pediatrics, without documented autoimmune disease, is a poorly characterized condition. Despite sporadic case reports, there is a notable absence of systematic studies or extensive case series that would allow a comprehensive understanding of this entity. Genetic mutations have emerged as factors linking LIP to immunodeficiencies and immune dysregulation, specifically mutations in genes such as COPA, CTLA-4, STAT3, TMEM173, and LRBA (18–22). In these patients, a clear correlation between autoimmunity and common variable immunodeficiency has been established.

In 2020, a seminal international case series involving 13 patients was published, representing the most robust account of LIP in pediatric patients to date. This series delineated a chronic clinical course marked by high morbidity and mortality rates, often accompanied by coexisting follicular bronchiolitis (23). Furthermore, the authors identified immunological dysregulation characterized by circulating autoantibodies and genetic studies revealing mutations in the COPA gene (18, 23). In our patient, the genetic study was performed by massively parallel single exome sequencing, analyzing coding and splicing regions. Three heterozygous variants were found in the SCNN1B, FCHO1 and IL7R genes.

The variant found in the SCNN1B gene was not described in the HGMD or NCBI ClinVar databases (accession number dbSNP rs372132399). The SCNN1B gene is a non-voltage-gated, amiloride-sensitive sodium channel that is involved in the control of fluid and electrolyte transport across the epithelium in a variety of organs. These channels are heteromeric complexes composed of three subunits: α, β, and γ. This gene encodes the β subunit and mutations are associated with pseudohypoaldosteronism type 1 (PHA1) and Liddle syndrome. Pathogenic mutations in this gene cause idiopathic bronchiectasis (IB), which presents as a progressive lung disease characterized by chronic bronchial dilatation and destruction of the bronchial walls in the absence of any underlying cause such as post-infectious disease, aspiration, immunodeficiency, congenital abnormalities and ciliary abnormalities (24).

The variant found in the FCHO1 gene is described in NCBI ClinVar as variant of uncertain significance and not described in HGMD (dbSNP accession number rs139967668). FCHO1 codes for a protein that is involved in forming and maturing clathrin vacuoles, which are a major route for internalizing cell surface proteins and molecules by endocytosis. Type 76 autosomal recessive immunodeficiency is caused by pathogenic mutations in this gene (24–30).

The variant found in the IL-7R gene has not been described in the HGMD database and is described in the NCBI ClinVar database as a variant of uncertain significance (dbSNP accession number rs753614229). The interleukin 7 (IL7) receptor is encoded by the IL7R gene. This protein plays an important role in VDJ binding during lymphocyte development. Defects in this gene may be associated with severe combined immunodeficiency (SCID) (24–30).

Although these heterozygous variants have not been reported to be aetiological for LIP, nor are they diagnostic for congenital immunodeficiency in the patient, in our opinion they are associated with severe lung damage. No alterations suggestive of SCID, type 76 autosomal recessive immunodeficiency, Liddle syndrome or PHA 1 were documented in the patient. The pathophysiology of LIP is unknown, dysregulation of the immune system has been shown to be important, as in some cases LIP may precede the development of malignancy. It has been suggested that abnormal activation of immune cells may be a factor in LIP (1, 2, 5, 31). One postulation is that abnormal activation of immune cells, triggered by either an infectious or unidentified stimulus, leads to an altered immune response. Furthermore, an immunogenetic basis has been postulated, with potential associations involving alterations in HLA-DR5, although this correlation has predominantly been described exclusively in adult cases (3, 31).

In our patient, HTLV-1 positivity was confirmed by Western blot, but no malignancy, autoimmunity, or opportunistic infections were documented. We think that this infection alone does not explain the severe lung damage found in the patient, although it may have been an associated or precipitating factor in the disease. Furthermore, although some cases of LIP with HTLV-1 have been described in adult patients (32–34), no clear relationship has been established as with HIV and Epstein-Barr virus.

In the case series published by Ingara et al. in a pediatric population in southwestern Colombia with HTLV-1 positive, they found nutritional deficiencies, skin involvement, opportunistic diseases, autoimmune and/or chronic inflammatory diseases, polymyositis, and pulmonary involvement as clinical manifestations. The latter was found in half of the patients, but bronchiectasis was related to the co-diagnosis of opportunistic infections by M. tuberculosis and Aspergillus, which were not documented in our patient (35).

The diagnosis of LIP in pediatrics relies on a combination of clinical and histopathological criteria (1, 3, 10, 16, 23). Chronic disease progression, associated with distinct radiological findings like a reticulonodular pattern, nodular changes, recurrent altering consolidations, and bronchiectasis, can guide suspicion for accurate diagnosis (1, 10, 11, 36). In the present case, the diagnosis was difficult because it was not possible to follow the patient over time to demonstrate chronic progression, as the patient was first admitted to our department with clear signs of advanced lung disease with severe functional impairment without a clear cause. To confirm or exclude the causes of severe chronic lung disease, histopathological review and a multidisciplinary approach were required to diagnose the patient with LIP. Lung transplantation was proposed as the last treatment option, as the patient was found to have severe pulmonary function impairment that was not improving with established treatment.

Due to the rarity of LIP without HIV infection in pediatric patients, studies with more robust cohorts and case reports with genetic studies will be helpful to increase knowledge of disease-related genetic alterations, thus improving diagnostic strategy, early detection, and appropriate intervention for patients with LIP. We have documented heterozygous variants in the SCNN1B, FCHO1, and IL7R genes in the patient, which may be associated with the severity of her disease. These variants have not been previously associated with NIL in our population.

## Conclusion

We have presented a case of a pediatric patient with LIP without HIV infection with HTLV-1 positivity, showcasing severe pulmonary involvement. No autoimmune disease, polymyositis, myelopathy, or opportunistic infections were found and there were no abnormalities in cellular and humoral immunity. Massively parallel single exome sequencing with analysis of coding and splicing regions found heterozygous variants in the SCNN1B, FCHO1, and IL7R genes. Recognition of these variants suggests their potential relevance, particularly in underrepresented populations like ours. The approach and diagnosis of LIP without HIV infection proves a challenge demanding a multidisciplinary clinical approach to explore immune dysregulation, autoimmunity, and inborn errors of immunity.

## Data Availability

The data supporting the findings of this report are restricted by the Ethics Committee of Fundación Valle del Lili to uphold patient privacy and confidentiality. Access to the patients medical history data is overseen by MOH and authorized co-investigators who meet the necessary criteria for accessing confidential data. Requests for data access should be directed to the designated responsible parties mentioned above, ensuring adherence to privacy and ethical guidelines.
